# The role of disease duration in the use of complementary and integrative medicine for cancer-related fatigue: a cross-sectional study

**DOI:** 10.1007/s00520-025-09367-z

**Published:** 2025-03-20

**Authors:** Yağmur Artan, Gökhan Sezgin, İrem Bulut, Yasemin Yildirim

**Affiliations:** 1 Faculty of Health Sciences, Nursing Department, Ardahan University, Merkez, Post Box 75000, Ardahan, Turkey; 2 Bone Marrow Transplantation Unit, İzmir City Hospital, İzmir, Turkey; 3 Oncology Unit, İzmir Acıbadem Kent Hospital, İzmir, Turkey; 4 Faculty of Nursing, Internal Medicine Nursing, Ege University, İzmir, Turkey

**Keywords:** Cancer, Complementary therapies, Fatigue, Integrative medicine, Nursing

## Abstract

**Purpose:**

This study aimed to assess the use of complementary and integrative medicine among cancer patients in Turkey for managing fatigue and to examine the moderating effect of disease duration on the relationship between fatigue levels and patients’ attitudes toward complementary and integrative medicine.

**Methods:**

A cross-sectional study was conducted with 231 cancer patients recruited from a chemotherapy center in western Turkey. Participants completed sociodemographic forms, the Holistic Complementary and Alternative Medicine Questionnaire, and the Visual Analog Scale for Fatigue. Data were analyzed using linear regression and moderation analysis.

**Results:**

Among the participants, 38.5% reported using complementary and integrative medicine to manage fatigue, with biologically based treatments such as vitamin supplements and herbal tea being the most common. Regression analysis showed that younger age (*p* < 0.05), longer disease duration (*p* = 0.005), absence of complementary and integrative medicine-related complications (*p* < 0.001), and higher fatigue levels (*p* < 0.001) were significant predictors of positive attitudes toward complementary and integrative medicine. Additionally, disease duration moderated the relationship between fatigue levels and attitudes toward complementary and integrative medicine, with longer disease duration strengthening the association (*B* =  − 0.269, SE = 0.078, *p* = 0.001).

**Conclusion:**

The findings suggest that fatigue and prolonged disease duration significantly influence cancer patients’ attitudes toward complementary and integrative medicine. Given the increasing use of complementary and integrative medicine, healthcare professionals should provide evidence-based guidance to ensure its safe and effective use. Future research should explore the long-term effects of complementary and integrative medicine on cancer-related fatigue.

**Relevance to clinical practice:**

This study highlights the importance of addressing cancer-related fatigue and recognizing the growing use of complementary and integrative medicine among cancer patients.

**Patient or public contribution:**

Patients were invited to complete questionnaires. Investigators explained the study’s objectives and content and addressed any concerns raised during data collection.

## Introduction

Cancer remains one of the leading causes of death worldwide. In 2022, nearly 20 million new cases were diagnosed, including nonmelanoma skin cancers, and the disease accounted for approximately 9.7 million deaths [1]. Current estimates suggest that one in five individuals will develop cancer during their lifetime, with around one in nine men and one in twelve women dying from the disease. Beyond its life-threatening nature, cancer and its treatments have a profound impact on patients’ quality of life, often causing a range of distressing symptoms [2].

Cancer-related fatigue (CRF) is a prevalent and debilitating symptom experienced by cancer patients. It is characterized by a persistent physical, emotional, and cognitive exhaustion that is disproportionate to recent activity and interferes with daily life. Unlike typical fatigue, CRF is more severe, does not improve with rest, and can significantly reduce patients’ quality of life. Studies show that most cancer patients experience some degree of fatigue during their illness [3]. Research indicates that approximately 40% of patients report fatigue at diagnosis, with prevalence increasing to 90% during radiation therapy and 80% during chemotherapy [4]. Although the exact causes of CRF are not fully understood, it is believed to result from both the disease itself and its treatments, including surgery, chemotherapy, and radiation therapy [5].

The etiology of CRF is multifactorial, involving both the disease itself and its treatments. Contributing factors may include anemia, sleep disturbances, pain, emotional distress, and metabolic changes. Despite its high prevalence and significant impact, CRF is often underreported and undertreated, highlighting the need for effective management strategies [6].

CRF affects patients physically, emotionally, and cognitively, often disrupting their daily lives. Even after treatment, many individuals struggle to return to their normal activities [7]. Its impact extends beyond patients, placing significant stress and anxiety on their families. Despite its high prevalence and debilitating effects, CRF is often underreported by patients, underestimated by healthcare providers, and inadequately treated [8]. Symptoms can arise during or after treatment and may persist for extended periods, ranging from 2 weeks to as long as 5 years [5].

Pharmacological strategies for managing CRF include psychostimulants, phytotherapeutics, antidepressants, corticosteroids, and growth factors. Other agents, such as erythropoietin-stimulating agents (ESA), dexmethylphenidate, modafinil, L-carnitine, and American ginseng, are also used [9]. However, due to the limited effectiveness and potential side effects of drug-based treatments, non-pharmacological approaches are increasingly preferred [10]. These alternative methods include exercise, psychosocial interventions, bright light therapy, yoga, dietary modifications, acupuncture/acupressure, and sleep therapy [11]. In the absence of a standardized medical treatment for CRF, many patients turn to CIM, believing it to be safer than conventional therapies [5, 12].

In recent years, interest in CIM for managing CRF has grown [13]. CIM includes a variety of practices and products that are not traditionally part of standard medical care, often emphasizing holistic well-being [14]. Evidence-based CIM approaches, such as mind–body interventions, acupuncture, and exercise programs, are increasingly being integrated into conventional cancer care to support patients throughout their treatment journey [15, 16]. However, the safety and effectiveness of many CIM treatments remain insufficiently studied, highlighting the need for further research [17].

Several factors influence cancer patients’ adoption of CIM, including sociodemographic characteristics, cultural beliefs, and the desire to take a more active role in their care. Understanding these factors is essential for healthcare providers to deliver patient-centered care and address potential barriers to effectively integrating CIM into cancer treatment plans [18].

Globally, CIM usage among cancer patients varies widely, ranging from 16.3 to 79%. Prevalence rates differ by regions, with reports indicating 49.3% in Asia [15], 51.8% in Iran [2], 54% in Sweden [19], 72.3% in Italy [20], and 79% in Norway [21]. In Turkey, CIM use is reported to range between 16.3 and 48% [22, 23].

Although the use of CIM among cancer patients is increasing, comprehensive research on its role in managing cancer-related fatigue remains limited, particularly in Turkey. Most existing studies focus on the general prevalence of CIM use rather than its association with fatigue levels and disease duration [18–23]. This knowledge gap restricts healthcare providers’ ability to offer evidence-based guidance on using CIM for fatigue management. Moreover, while previous research has highlighted the potential benefits of CIM, the influence of disease duration on patients’ attitudes toward CIM remains largely unexplored.

This study aims to assess the use of CIM among cancer patients in Turkey for managing fatigue and examine the moderating effect of disease duration on the relationship between fatigue levels and patients’ attitudes toward CIM. By exploring these factors, the study seeks to provide new insights into how CIM use is influenced by disease duration and fatigue severity, contributing to a more comprehensive understanding of CIM in cancer care.

## Materıal and methods

### Theoretical framework

This study is grounded in Dossey’s Theory of Integral Nursing, which proposes a holistic, integrative, person-centered approach to health care. Integral nursing provides a mind–body-spirit perspective as a comprehensive framework for patients and caregivers. According to Dossey, healing is a natural, multidimensional process that considers the connections between individuals, their environment, and the systems of which they are a part [24]. The meta-paradigm of the theory (i.e., human, environment, health, nursing) aligns well with the context of cancer care. It emphasizes the dynamic and evolving relationships between nurses, patients, and their surroundings. This integral perspective serves as a foundational lens for understanding how holistic, patient-centered interventions can enhance quality of life and support recovery in individuals with cancer.

The specific research hypothesis is as follows: (1) Sociodemographic characteristics may influence cancer patients’ attitudes towards CIM; (2) Fatigue levels may be positively associated with cancer patients’ attitudes towards CIM; (3) Disease duration may moderate the relationship between fatigue levels and cancer patients’ attitudes toward CIM.

The answers to the following questions were sought in the study:

1. What factors influence cancer patients’ use of CIM for managing cancer-related fatigue?

Secondary Research Questions:

2. Does the severity of fatigue impact cancer patients’ attitudes toward CIM?
3. Does disease duration moderate the relationship between fatigue levels and cancer patients’ attitudes toward CIM?

### Study design

This cross-sectional study employed a paper-based, face-to-face survey to investigate the complementary and integrative medicine practices used by cancer patients in Turkey to manage fatigue. Data collection took place between September 2023 and January 2024.

### Sampling and recruitment

Participants were recruited from a chemotherapy center at a university hospital in western Turkey. Since reaching the entire population was challenging, a probability sampling method was used for sample selection. The Holistic Complementary and Alternative Medicine Questionnaire (HCAMQ) average score was used in the power analysis, conducted using G*Power software, with an effect size of 0.3, an α level of 0.05, and a power of 0.95. Based on these parameters, a minimum of 220 patients was required to achieve an 80% response rate. To account for potential dropouts, 240 participants were recruited, all of whom completed the questionnaire. However, nine invalid paper-based questionnaires were excluded, resulting in a final sample of 231 valid responses. The overall response rate was 96.0%.

As a result, 231 cancer patients were included in the study. The inclusion criteria were as follows: (1) age > 18 years, (2) ability to speak and understand Turkish, and (3) diagnosis of cancer and undergoing chemotherapy for at least 6 months. Patients who did not experience fatigue were excluded from the study. Data were collected using the “Sociodemographic form,” “Holistic Complementary and Alternative Medicine Questionnaire (HCAMQ),” and “Visual Analogue Scale-Fatigue (VAS-F).”

### Data collection

The researchers collected data for this study between September 2023 and January 2024 using face-to-face, paper-based surveys conducted at a chemotherapy center in a university hospital in western Turkey. Before administering the questionnaire, the researchers provided patients with detailed information about the study’s objectives, procedures, and ethical considerations. After obtaining informed consent, the questionnaire was distributed in a designated private consultation room within the hospital. Patients completed the survey at the hospital before their chemotherapy sessions, ensuring that fatigue levels were assessed in a controlled setting. During data collection, the researchers were present to answer questions, clarify instructions, and ensure that participants completed the survey independently, without external influence. Each participant took approximately 15 min to complete the survey. By structuring the data collection process in this manner, we aimed to maintain consistency in administering the questionnaire and minimize potential biases related to timing and setting.

### Measurements

#### Sociodemographic form

This description refers to a research form developed by the researcher based on the literature [10, 16, 17]. The form includes 20 questions related to sociodemographics (age, gender, body mass index (BMI), marital status, etc.) and CIM usage of the patients (CIM methods, reasons for not using CIM, complications while using CIM, etc.).

#### Holistic complementary and alternative medicine questionnaire (HCAMQ)

The scale developed by Hyland et al. for rheumatology outpatients in the UK [25] was adapted into Turkish by Erci [26]. The scale measures beliefs about CIM and consists of 11 items, each rated on a six-point Likert scale ranging from 1 (strongly agree) to 6 (strongly disagree). All items are positively worded. The total possible score ranges from 11 to 66, with lower scores indicating more positive attitudes toward CIM use. The scale has two sub-dimensions: complementary and alternative medicine (CAM) (2, 4, 6, 8, 10, 12 items) and holistic health (HH) (1, 3, 5, 7, 11 items). The total score is calculated by summing the values assigned to all items. The Cronbach’s alpha coefficient for the entire scale is 0.72, while in this study, it was found to be 0.89, indicating high internal consistency.

#### Visual analogue scale-fatigue

The Visual Analogue Scale for Fatigue (VAS-F) was used to assess patients’ fatigue levels. Patients rated their fatigue on a 0 to 10 scale, where 0 indicated no fatigue, and 10 represented severe fatigue. Each patient selected a point on the scale that best reflected their fatigue level.

### Data analysis

**Fig. 1:**
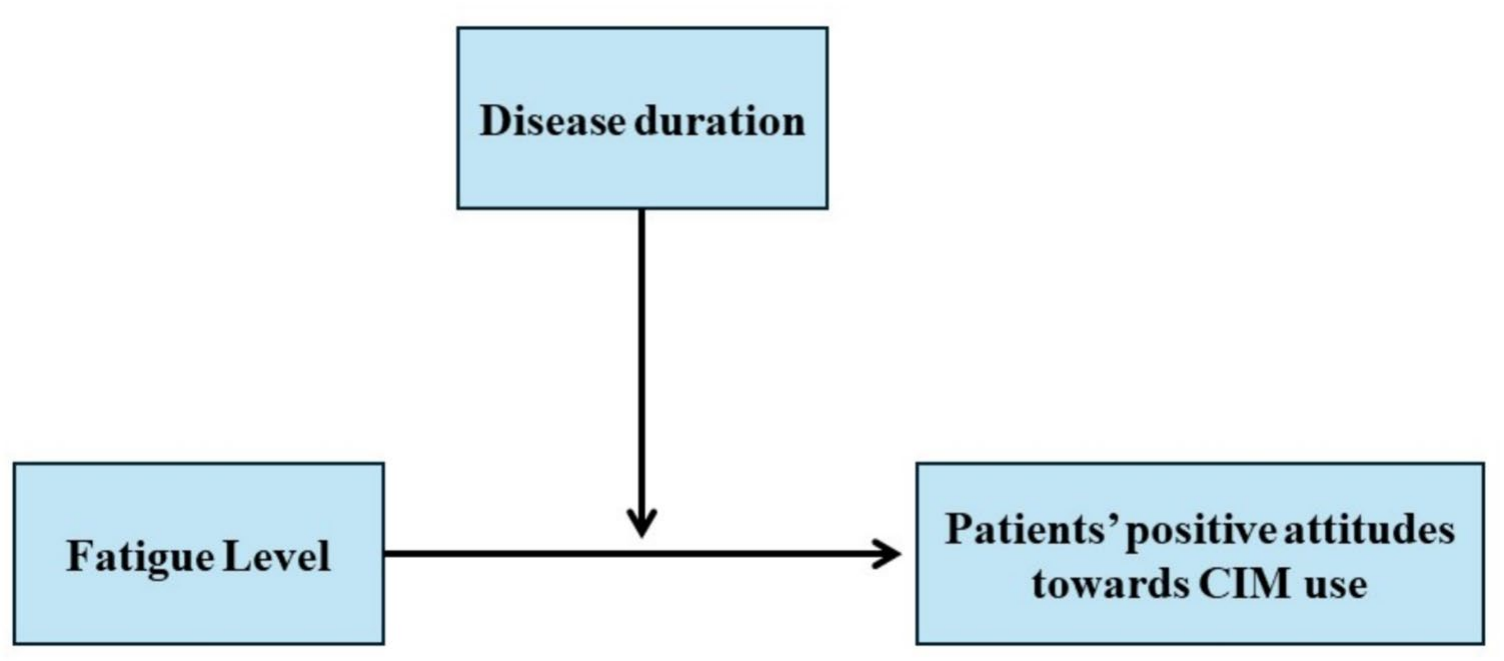
Moderation model of the relationships of the study variables

Statistical analyses were undertaken using the SPSS version 23 package program. Based on normality, sociodemographics and disease characteristics are reported as means (standard deviations [SD]) for parametric and medians (ranges) for non-parametric data. An independent samples *t*-test and chi-squared test were utilized to analyze the differences in sociodemographic variables and CIM use. Univariate linear regression analysis was used to examine the relationship between fatigue levels and cancer patients’ attitudes toward CIM. Multiple linear regression analysis identified the significant factors influencing cancer patients’ attitudes toward CIM use. The moderation model illustrating the relationships among study variables is presented in Fig. 1. Moderation effects between fatigue levels and attitudes toward CIM were analyzed using the SPSS PROCESS macro (Model 1) [27]. Bootstrapping was used with a sample size of 5000, and indirect effects were considered significant if the 95% confidence interval (CI) did not include 0. A *p*-value of less than 0.05 was considered statistically significant for all analyses.

### Ethical considerations

Ethical permission was approved by the Medical Research Ethics Committee of Ege University (decision date and number: 24.08.2023, 23-8 T/57). The study protocol was designed in accordance with Good Clinical Practice and conducted in compliance with the Declaration of Helsinki.

## Results

### Participants’ and disease-related characteristics

The sociodemographic and clinical characteristics of participants are shown in Table 1. A total of 231 cancer patients were included in the study. The mean age of the sample was 54.23 ± 13.11 years, and 63.2% of patients were female. The mean duration of the disease was 5.82 ± 2.79 years. More than half of the participants were married (74.5%), diagnosed with breast cancer (33.3%), had a chronic disease (51.9%), and had no metastasis (66.7%), and 40.3% were primary school graduates. A significant difference was found between CIM users and non-users in terms of disease duration (*p* = 0.004), VAS-F scores (*p* < 0.05), and HCAMQ scores (*p* = 0.000).

**Table 1:** Sociodemographic and disease-related characteristics of users and non-users of the complementary and integrative medicine (CIM)

Variables		Mean ± SD/no. (%)			
		Total (*n* = 231)	CIM users (*n* = 89, 38.5%)	Non-users of CIM (*n* = 142, 61.5%)	*p*
Gender	Female Male	146 (63.2) 85 (36.8)	59 (66.3) 30 (33.7)	87 (61.3) 55 (38.7)	0.441^*^
Education level	Primary school High school University	93 (40.3) 67 (29.0) 71 (30.7)	36 (40.4) 28 (31.5) 25 (28.1)	57 (40.1) 39 (27.5) 46 (32.4)	0.729^*^
Marital status	Single Married	59 (25.5) 172 (74.5)	23 (25.8) 66 (74.2)	36 (25.4) 106 (74.6)	0.934^*^
Income level	Less than expenditure Equal to expenditure Higher than expenditure	67 (29.0) 143 (61.9) 21 (9.1)	28 (31.5) 55 (61.8) 6 (6.7)	39 (27.5) 88 (62.0 15 (10.5)	0.554^*^
Living area	Village Town City	11 (4.8) 72 (31.2) 148 (64.1)	2 (2.2) 28 (31.5) 59 (66.3)	9 (6.3) 44 (31.0) 89 (62.7)	0.361^*^
Family history of cancer	Yes No	*145 (62.8)* *86 (37.2)*	57 (64.0) 32 (36.0)	88 (62.0) 54 (38.0)	0.751^*^
Type of cancer	Breast Hematological Gastrointestinal Respiratory system Genitourinary Other(s)	77 (33.3) 52 (22.5) 39 (16.9) 17 (7.4) 18 (7.8) 28 (12.1)	31 (34.8) 20 (22.5) 14 (15.7) 5 (5.6) 12 (13.5) 7 (7.9)	46 (32.4) 32 (22.5) 25 (17.6) 12 (8.5) 6 (4.2) 21 (14.8)	0.109^*^
Metastasis	Yes No	77 (33.3) 154 (66.7)	31 (34.8) 58 (65.2)	46 (32.4) 96 (67.6)	0.702^*^
Chronic disease	Yes No	130 (51.9) 101 (48.1)	45 (50.6) 44 (49.4)	75 (52.8) 67 (47.2)	0.738^*^
Age (*y*)		54.23 ± 13.11	53.48 ± 11.85	54.70 ± 13.86	0.492^**^
Disease duration (year)		5.82 ± 2.79	6.55 ± 3.31	5.37 ± 2.30	0.004^**^
VAS-F		5.96 ± 1.44	6.23 ± 1.34	5.80 ± 1.47	0.026^**^
Holistic Complementary and Alternative Medicine Questionnaire (HCAMQ)		20.46 ± 7.16	15.80 ± 2.91	23.38 ± 7.48	0.000^**^

*Chi-squared test; **Independent *t*-test

### Patients’ use of complementary and integrative medicines

Among the cancer patients in this study, 38.5% use CIM (Table 1). The most commonly used CIM methods were biologically based treatments, such as vitamin supplements (16.8%), mind–body techniques, such as relaxation/exercises (33.7%), and energy treatment methods, such as reiki (4.5%) (Table 2). More than half of the patients avoided using CIM, believing that these methods could be hazardous. Additionally, the study found that 68.5% of the patients felt better after using CIM, 92.1% did not experience any complications from CIM use, 48.4% received CIM-related information primarily from relatives or other patients, and 78.7% recommended using CIM (Table 3).

**Table 2:** Patients’ use of complementary and integrative medicines (CIM)

CIM method		CIM users (*n* = 89), no. (%)
Biologically-based treatment methods (*n* = 127)	Vitamin supplement Herbal tea Bone broth Grape molasses Probiotics Bee gum Honey Tahina Curcumin Nuts Black cumin Tutsan Fish oil Magnesium Garlic Ginseng Zinc Olive oil Asparagus	15 (16.8) 14 (15.7) 14 (15.7) 12 (13.4) 10 (11.2) 10 (11.2) 9 (10.1) 9 (10.1) 7 (7.8) 7 (7.8) 4 (4.5) 4 (4.5) 3 (3.3) 3 (3.3) 2 (2.2) 1 (1.1) 1 (1.1) 1 (1.1) 1 (1.1)
Mind–body methods (*n* = 41)	Relaxation/exercises Meditation Yoga Music therapy	30 (33.7) 7 (7.8) 2 (2.2) 2 (2.2)
Energy treatment methods (*n* = 4)	Reiki	4 (4.5)

**Table 3:** Patients’ complementary and integrative medicines (CIM)-related characteristics

Variables		No. (%)
CIM use	Yes No	89 (38.5) 142 (61.5)
Reasons for not using CIM (*n* = 142)	I don’t believe in the effects of CIM methods I think CIM methods are more hazardous	50 (35.2) 92 (64.8)
Using CIM since… (*n* = 89)	Before diagnosis After diagnosis	43 (48.3) 46 (51.7)
Effect of using CIM (*n* = 89)	It wasn’t effective I felt better than before It only relaxed me psychologically	11 (12.4) 61 (68.5) 17 (19.1)
Complications while using CIM (*n* = 89)	Yes No	7 (7.9) 82 (92.1)
Recommendation to others for CIM use (*n* = 89)	Yes No	70 (78.7) 19 (21.3)
Source of information on CIM use (*n* = 89)	Health professionals Relatives/other patients Internet/social media	23 (25.8) 43 (48.4) 23 (25.8)

### Examination of factors affecting HCAMQ score according to linear regression analysis

The linear regression analysis conducted in this study developed two models. In Model 1, the explanatory power of fatigue level on the HCAMQ score alone was 23.4% (Model 1; *β* =  − 2.418, Adjusted *R*^2^ = 0.234, *p* = 0.000). In Model 2, the inclusion of sociodemographic and health-related characteristics increased the explanatory power to 41.6% (Adjusted *R*^2^ = 0.416, *F* = 7.951, *p* = 0.000). The analysis revealed that advanced age was a positive predictor of HCAMQ scores (*p* < 0.05), while disease duration and the absence of complications from CIM use were negative predictors (*p* = 0.05) (Table 4). These findings suggest that positive attitudes toward CIM tend to decline with age. However, patients with higher fatigue levels, longer disease duration, and no complications from CIM use were more likely to have favorable attitudes toward incorporating CIM into their care.

**Table 4:** Examination of factors affecting HCAMQ score according to linear regression analysis

Models	*B*	Beta	*t*	*p*	Adjust *R*^2^	Model *p*	95% confidence interval	
							Lower bound	Upper bound
Model 1								
Constant VAS-F	34.899 − 2.418	- − 0.487	15.470 − 8.443	0.000 0.000	- 0.234	*F* = 71.282 0.000	31.433 − 2.982	38.364 − 1.854
Model 2								
Constant Age Duration of ilness Lacking complications	22.967 0.072 − 0.392 − 4.709	- 0.293 − 0.446 − 0.437	9.128 2.233 − 3.238 − 5.106	0.000 0.028 0.002 0.000	0.416	*F* = 7.951 0.000	17.959 0.008 − 0.633 − 6.544	27.976 0.136 − 0.151 − 2.873

### Disease duration as a moderator between fatigue level and HCAMQ score

Table 5 presents the conditional effect of disease duration. The relationship between the fatigue level and HCAMQ score has a negative effect at the low, medium, and high levels of disease duration (*B* =  − 0.431, SE = 0.087, *p* = 0.000), (*B* =  − 0.269, SE = 0.078, *p* = 0.001), and (*B* =  − 0.106, SE = 0.103, *p* > 0.05), respectively. According to this result, disease duration strengthens the relationship between fatigue levels and HCAMQ scores.

**Table 5:** Conditional effects of disease duration on relationship between fatigue level and HCAMQ score

Disease duration	*β*	SE	*t*	*p*	95% confidence interval	
					Lower bound	Upper bound
Low (one *SD* below mean) Average (at the mean) High (one *SD* above mean)	− 0.431 − 0.269 − 0.106	0.087 0.078 0.103	− 4.967 − 3.424 − 1.034	0.000 0.001 0.302	− 0.602 − 0.423 − 0.308	− 0.260 − 0.114 0.096

**Fig. 2:**
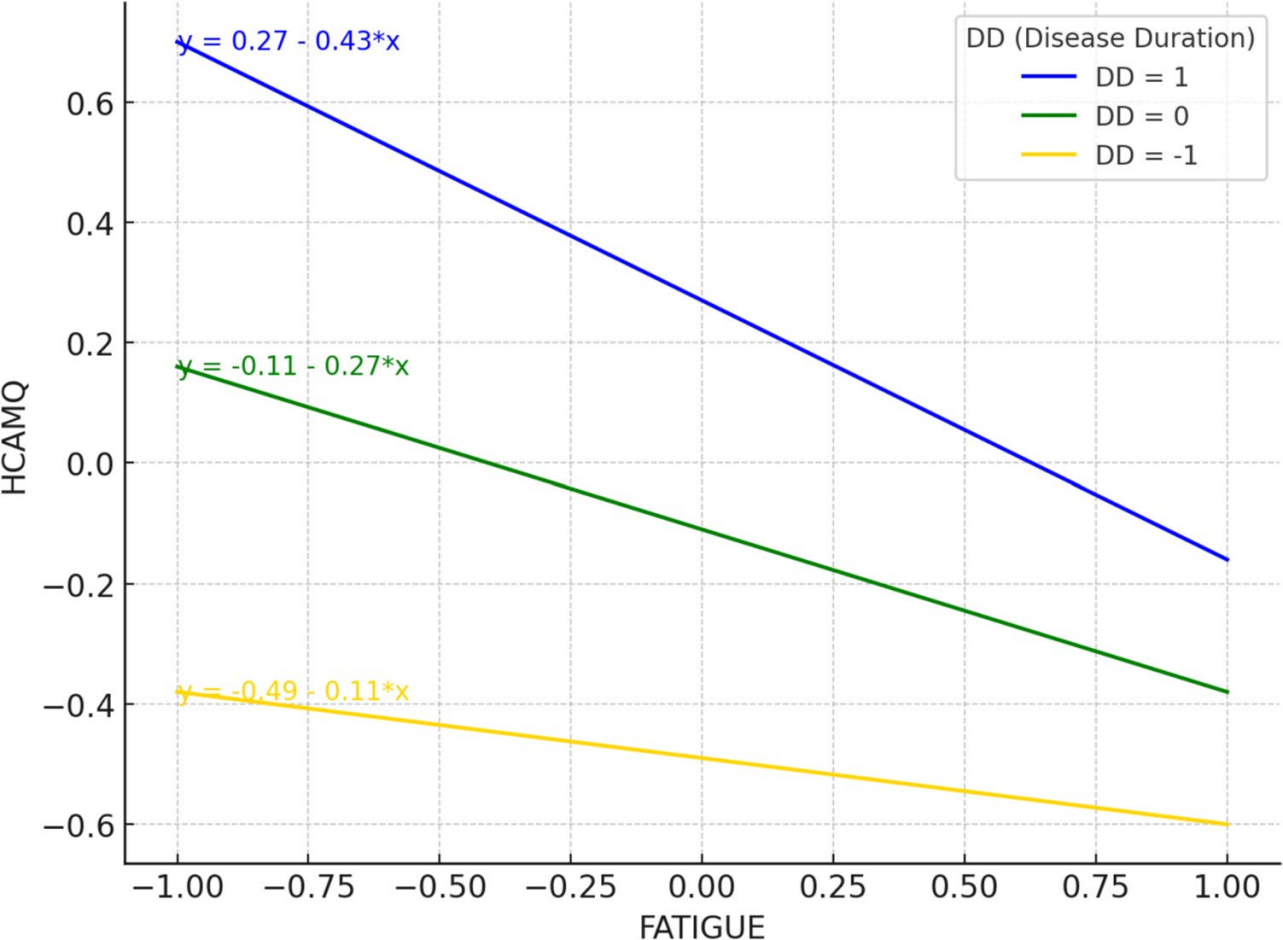
Moderating effect of disease duration at different levels on the relationship between fatigue level and HCAMQ score

Figure 2 shows a better understanding of this conditional effect. The relationship between the fatigue level and HCAMQ score presented a negative correlation. In other words, as fatigue levels increase, cancer patients’ positive attitudes toward CIM also become stronger. Additionally, longer disease duration amplifies this effect, leading to lower HCAMQ scores, which reflect greater acceptance of CIM. These results highlight the significant moderating role of disease duration in the relationship between fatigue levels and HCAMQ scores.

## Discussion

Cancer-related fatigue is one of the most common and distressing symptoms experienced by cancer patients, significantly affecting their quality of life. CRF often goes underreported and undertreated, leading many patients to seek CIM as an alternative or supportive approach. However, the factors influencing patients’ attitudes toward CIM and the role of disease duration in this relationship have not been thoroughly explored. This study aimed to address this gap by examining the CIM practices used by cancer patients in Turkey to manage fatigue and exploring whether disease duration moderates the association between fatigue levels and attitudes toward CIM. Understanding these relationships is crucial for developing targeted interventions that help patients navigate safe and effective CIM use in cancer care.

This study is the first to investigate CIM use for managing cancer-related fatigue in a Turkish context. Findings revealed that 38.5% of cancer patients used at least one CIM method, with biologically based therapies being the most commonly preferred. Younger age, longer disease duration, absence of CIM-related complications, and higher fatigue levels were identified as significant predictors of more positive attitudes toward CIM.

We found that 38.5% of participants used at least one CIM modality to manage fatigue. Due to the limited research specifically addressing CIM use for fatigue management, we compared our findings with studies examining CIM use for other cancer-related symptoms, such as taste changes and nausea. Research on general CIM use among cancer patients reports prevalence rates ranging from 16.3 to 79.0% [12, 19, 22, 23]. Differences in CIM usage rates may be influenced by socio-cultural factors, variations in sample age, accessibility and cost of treatments, differences in cancer-related symptoms, and the specific CIM modalities studied.

In this study, the most commonly used CIM modalities were biologically based treatments, such as vitamin supplements and herbal teas. Research on CIM use among cancer patients consistently highlights biologically based treatments as the most frequently used approach [12, 20, 21, 23]. Herbal products, in particular, are often perceived as natural, free from side effects, safe for long-term use, and effective in alleviating symptoms. Additionally, they are affordable and easily accessible. Our findings also indicate that relatives and other patients were the primary sources of information on CIM use (48.4%). This suggests that peer recommendations may contribute to the widespread use of herbal medicines and supplements among cancer patients. Interestingly, only a small proportion of the CIM modalities studied were used by participants. Manipulative treatments, such as acupressure and acupuncture, were not utilized at all, while mind–body techniques, including meditation, yoga, and hypnosis, were used infrequently. This limited adoption may be influenced by cultural factors in Turkey, where practices such as meditation and yoga, which are not traditionally rooted in the local culture, are less widely accepted [28].

This study found that patients primarily obtained information on CIM methods for managing fatigue from other patients and relatives. Research indicates that CIM knowledge is often acquired through peer recommendations rather than scientific sources [29, 30]. This finding suggests that patients rely on informal and potentially unreliable sources, basing their decisions on personal experiences rather than evidence-based information. Therefore, healthcare professionals, particularly nurses, should assess patients’ CIM practices and evaluate the sources of their information [31]. Providing accurate, comprehensive, and balanced information on both conventional and alternative treatments should be a key responsibility of healthcare providers and educators [32]. However, studies indicate that healthcare professionals in many countries lack sufficient knowledge of CIM modalities, highlighting the need for better training and awareness in this area [33].

A study conducted with adolescent cancer patients found that the most common reason for not using CIM was a lack of information from healthcare professionals [34]. As the use of CIM among cancer patients is expected to increase in the future, oncology healthcare providers should enhance their knowledge of CIM therapies to help minimize potential risks and adverse effects associated with their use [35]. Furthermore, it is recommended that healthcare professionals regularly monitor cancer patients’ CIM use and assess them for possible side effects or safety concerns.

The key predictors of CIM use for managing fatigue were VAS-F score, age, disease duration, and the absence of complications. Our findings align with existing literature, which suggests that greater knowledge of CIM and the absence of complications increase trust in CIM and lead to higher usage rates among patients [12, 36]. We also found a statistically significant relationship between positive attitudes toward CIM use and younger age, consistent with findings from international surveys [8, 36]. This trend may indicate higher health literacy among CIM users. However, research on the relationship between age and CIM use remains inconclusive. While some studies have reported no association [23], others suggest that older patients are more likely to use CIM [12]. These discrepancies may be due to regional differences in study populations.

In this study, the fatigue level emerged as a key predictor of CIM use. Similarly, research on cancer survivors has identified fatigue as a significant factor influencing the use of integrative therapies [37]. It is well established that CRF is highly prevalent among patients undergoing chemotherapy and radiotherapy [38]. There is a broad consensus that addressing CRF through a single intervention or medication is challenging due to its multiple underlying causes and the diverse clinical presentations observed in patients [39]. As a result, many patients turn to CIM approaches, seeking symptom relief and a proactive way to manage their health. Studies suggest that therapies such as targeted exercise therapy [40], reflexology [41], acupuncture [42], combined hypnosis and cognitive behavioral therapy [38], micronutrient supplementation [43], and herbal medicine [44] may help alleviate fatigue and improve overall well-being. Furthermore, cancer survivors experiencing severe fatigue may be particularly motivated to explore CIM, especially when conventional treatments fail to provide adequate relief [39]. These findings underscore the importance of supporting informed patient decisions and promoting safe CIM practices, particularly for those experiencing high levels of fatigue.

Variations in CIM use among cancer patients may be influenced by shifts in perception and coping mechanisms throughout their illness. In the early stages following diagnosis, patients may turn to alternative therapies as a way to regain a sense of control and take immediate action in response to uncertainty, leading to higher CIM use within the first year [45]. As the illness progresses, patients often become more aware of its chronic and unpredictable nature, which may intensify their search for relief, comfort, or holistic approaches that complement standard treatments. This shift may be driven by a growing awareness of the limitations of conventional therapies or an accumulating fear of mortality [46]. Consequently, prolonged illness may increase patients’ inclination toward CIM as a supplement to medical care, aligning with findings that longer disease duration is a significant predictor of CIM use [47].

Our findings reveal a negative correlation between fatigue levels and HCAMQ scores, indicating that as fatigue increases, patients develop more positive attitudes toward CIM. This trend is particularly evident in patients with longer disease duration, suggesting that chronic fatigue associated with prolonged illness increases openness to exploring CIM options. Previous research has shown that a higher symptom burden often motivates patients to seek complementary therapies as a way to regain control and enhance quality of life [44]. These findings are consistent with findings by Gok Metin et al. [45], who reported that prolonged illness and related fatigue drive patients to explore alternative therapies, likely due to a perceived inadequacy of conventional treatments in managing persistent symptoms. The cumulative fatigue associated with a long-term illness may thus encourage a re-evaluation of standard care and foster a more favorable view of CIM, as observed in the lower HCAMQ scores. These findings highlight the need for healthcare providers to recognize the changing attitudes toward CIM among cancer patients and to actively engage in discussions about safe, evidence-based integrative therapies as part of comprehensive patient care.

### Limitations and strengths

This study has several limitations. First, its cross-sectional design limits the ability to establish causal relationships between cancer-related fatigue, CIM use, and disease duration, as data were collected at a single time point. A longitudinal approach would offer deeper insights into how attitudes toward CIM and fatigue management evolve over the course of the disease. Additionally, reliance on self-reported data may introduce recall bias, potentially affecting the accuracy of reported CIM use and fatigue levels. Furthermore, because fatigue was assessed using the VAS, it was not possible to analyze its sub-dimensions. Future research should consider using comprehensive fatigue assessment tools to provide a more detailed evaluation.

Secondly, because the sample consists of cancer patients from a single chemotherapy center in western Turkey, the findings may not be generalizable to broader populations. Future research should include larger, more diverse samples from multiple regions to improve generalizability. Additionally, this study did not examine certain factors that may influence attitudes toward CIM, such as psychological well-being and support systems. Future studies should explore these aspects to provide a more comprehensive understanding of CIM use among cancer patients.

Despite its limitations, this study is among the first to examine CIM use for fatigue management among cancer patients in Turkey, addressing a previously under-researched area with important implications for patient-centered care. With a relatively large and representative sample of adult cancer patients, the findings are clinically relevant and offer valuable insights into how disease duration and fatigue levels influence attitudes toward CIM. This research provides a foundation for future studies exploring personalized, integrative approaches to managing cancer-related fatigue. Furthermore, the study highlights the moderating role of disease duration, underscoring the need for longitudinal research to develop more tailored and holistic cancer care strategies.

### Relevance to clinical practice

This study emphasizes the importance of addressing cancer-related fatigue and the increasing use of CIM among oncology patients. Healthcare professionals play a crucial role in guiding patients toward safe, evidence-based CIM practices, helping to reduce the risks associated with informal and potentially unreliable sources. Providing personalized education and consultations can empower patients to make informed decisions about their care. Collaborating with trained CIM practitioners and integrating CIM into oncology programs can enhance symptom management and improve patient satisfaction. For patients with prolonged illness and persistent fatigue, dynamic and personalized care plans are essential to address their changing needs and preferences effectively.

Additionally, healthcare professionals must expand their knowledge of CIM to provide more effective patient support. Continuing education programs should emphasize the safety, efficacy, and evidence base of CIM modalities, enabling informed discussions between patients and providers. This study also highlights the need for further research on culturally specific CIM practices and their effectiveness in managing cancer-related fatigue. Strengthening the evidence base will aid CIM’s safe and culturally relevant integration into oncology care.

## Conclusion

This study provides valuable insights into the use of complementary and integrative medicine (CIM) for managing cancer-related fatigue among patients in Turkey. The findings suggest that the relationship between fatigue levels and attitudes toward CIM is complex and context-dependent, with disease duration serving as a key moderating factor. Specifically, the results indicate that fatigue can both positively and negatively influence attitudes toward CIM, depending on the length of the illness. As more cancer patients turn to integrative approaches to manage their symptoms, it is essential for healthcare providers to facilitate informed discussions about CIM. By promoting safe, evidence-based practices, healthcare professionals can provide better support for patients navigating their treatment journeys, ultimately enhancing the quality of care and improving patient outcomes in cancer management.

## Data Availability

No datasets were generated or analysed during the current study.
